# The Application of Saline–Alkali-Tolerant Growth-Promoting Endophytic Bacteria for Enhancing the Saline–Alkali Tolerance of Alfalfa

**DOI:** 10.3390/biology15060474

**Published:** 2026-03-15

**Authors:** Muhammad Rahman Ali Shah, Lu Tang, Hao Zhou, Huiying Zheng, Yimeng Shi, Changhong Guo

**Affiliations:** Heilongjiang Provincial Key Laboratory of Molecular Cellular Genetics and Genetic Breeding, College of Life Science and Technology, Harbin Normal University, Harbin 150025, China; 2019731002@stu.hrbnu.edu.cn (M.R.A.S.); tanglu900816@hrbnu.edu.cn (L.T.); zhouhao08162025@163.com (H.Z.); huiying123451026@163.com (H.Z.); shiyimeng1026@163.com (Y.S.)

**Keywords:** alfalfa, saline–alkali conditions, endophytic bacteria, growth promoting

## Abstract

Saline–alkali stress is a severe abiotic factor that limits plant growth and development. Endophytic bacteria can improve plant tolerance to such stress through various mechanism, including osmoregulatory substance accumulation and antioxidant enzyme activity. In this study, four saline–alkali-tolerant endophytic strains, designated SYM-2, SYM-4, SYM-9, and SYM-15, were isolated from the roots of alfalfa grown in saline–alkali soil. Though 16S rDNA sequencing, morphological observations, and physiological–biochemical characterization, the strains were identified as closely related to *Bacillus cereus*, *B. thuringiensis*, *B. halotolerans*, and *Pantoea agglomerans*, respectively. These strains demonstrated the ability to produce 1-aminocyclopropane-1-carboxylate (ACC) deaminase, siderophores, and indole-3-acetic acid (IAA), as well as solubilizing phosphorus. Under saline–alkali conditions, inoculation with these strains significantly increased alfalfa growth parameters. Plant height increased by 4.07–33.90% and root length by 7.49–27.94%, and fresh and dry weight (both above and below ground) increased compared with the control. Strain SYM-15 showed the highest promoting effects, increasing plant height by 33.90%, root length by 27.94% and shoot dry weight by 59.26%. Additionally, root activity increased by 11.23–40%, proline content by 19.09–129–87%, and soluble protein by 7.71–42.49%, and the activities of catalase (CAT), peroxidase (POD), and superoxide dismutase (SOD) were significantly elevated across treatments. At the same time, inoculation reduced the levels of hydrogen peroxide (H_2_O_2_), superoxide anion (O_2_^−^), and malondialdehyde (MDA). Compared with the control and other treatments, including SYM-9, the peroxidase activity and superoxide dismutase activity of alfalfa significantly increased after the SYM-15 treatment, while hydrogen peroxide content, phosphorus content, and neutral detergent fiber and acid detergent fiber contents decreased (*p* < 0.05). Therefore, SYM-15 plays an important role in promoting growth and represents a promising, high-quality strain resource for the large-scale development of microbials aimed at improving alfalfa tolerance under saline–alkali conditions.

## Simple Summary

Soil salinity and alkalinity limit crop growth worldwide. Alfalfa, an important livestock feed, grows poorly in such soils. We isolated beneficial bacteria from alfalfa roots growing in saline–alkali soil in northeast China. Four bacterial strains were tested for their ability to help alfalfa grow under stress. These bacteria produced growth-promoting substances that stimulated root development and helped plants absorb nutrients. In pot and field experiments, the bacteria significantly improved plant height, root biomass, and overall yield. The best strain was SYM-15, which increased alfalfa yield by 34.5% and protein content by 13.3% while reducing fiber content. This research study shows that naturally occurring bacteria can be developed into environmentally friendly bio-fertilizers to improve crop production on marginal lands, contributing to sustainable agriculture.

## 1. Introduction

Soil salinization is a global environmental issue, affecting an estimated 932 million hectares of agricultural land worldwide, which represent about 20% of total cultivated land and 33% of irrigated agricultural land [1]. It poses a significant threat to agricultural ecosystem and crop productivity, leading to reduced land availability, degraded soil fertility, and ultimately limited global food production [2]. When soil salt content becomes excessive, water potential decreases due to increased salt concentration, which hinders water uptake by plant roots and triggers a series of physiological and biochemical responses. Moreover, in high-salinity environments, heavy metal ions in the soil can become more mobile through ion exchange or structural changes, subsequently entering plants via the roots and accumulating in tissues. This can lead to heavy metal toxicity, growth inhibition, and leaf yellowing [3]. High soil alkalinity can directly damage root cell structures and functions, leading to stunted development, abnormal morphology, and even root death [4]. Under strongly alkaline conditions, the solubility and availability of mineral elements are altered, limiting root uptake of phosphorus, iron, zinc, and other essential nutrients. This nutrient deficiency can lead to the wilting of above-ground leaves and impaired photosynthesis [5]. Saline–alkali soils represent an increasingly serious agriculture problem, reducing land availability, degrading soil fertility and productivity, shrinking arable areas, and ultimately limiting crop yields and planting ranges. Therefore, improving the utilization of saline–alkali land is crucial to maintaining the dynamic balance of arable land and achieving sustainable agricultural development.

Plant growth-promoting endophytes (PGPEs) are microorganisms that colonize the internal tissue of plants without causing apparent harm or disease symptoms and can be isolated from surface-disinfected plant tissue [6]. These bacteria spend at least part of their life cycle within host plants and can establish mutualistic relationship that benefit plant growth and health [7]. Endophytes play key roles in plant development, nutrient uptake, and oxidative stress tolerance, promoting plant growth through diverse mechanisms [8]. Numerous studies have reported beneficial plant–endophyte interactions, including plant growth promotion via nitrogen fixation, phosphorus solubilization, production of 1-aminocyclopropane-1-carboxylate (ACC) deaminase, synthesis of indole-3-acetic acid (IAA), secretion of siderophores, and emission of volatile organic compounds. PGPEs also enhance nutrient absorption and improve plant resistance to biotic and abiotic stresses, positioning them as integral components of plant systems and a key category of microbial resources. PGPEs perform two primary functions: they (1) supply essential energy and nutrients for plant growth and (2) enhance plant resilience through signal transduction pathways or the synthesis of beneficial metabolites [9]. Research has highlighted a strong association between endophytic bacteria and improved plant stress tolerance. PGPEs enhance plant growth and stress tolerance through multiple mechanisms, such as facilitating water and nutrient uptake, producing plant hormones and siderophores, regulating proline levels, and increasing antioxidant enzyme activity [10,11]. While numerous studies have demonstrated the potential of PGPB in mitigating stress in various crops, the majority have focused on neutral salt stress (NaCl) rather than alkaline stress (high pH combined with salinity). For instance, Lu et al. [12] isolated eight salt-tolerant endophytic bacterial strains from rice roots, among which strain D1 significantly improved rice growth, root development, and shoot development under saline–alkali stress, but they did not address alkaline conditions, where high pH additionally limits nutrient availability. Additionally, rice seedlings inoculated with strain D1 showed significant increases in proline, soluble protein, and chlorophyll contents. These findings underscore the potential of endophytic bacteria in enhancing plant stress tolerance. Similarly, Masmoudi et al. [13] demonstrated that salt-tolerant *Bacillus* sp. FMH2 alleviates salt stress in tomato, but the study only considered NaCl stress and not alkaline components. Strain FMH2 produces substantial amounts of siderophores, IAA, and various hydrolytic enzymes. Collectively, these traits promote tomato growth under salt stress by improving physiological performance and enhancing antioxidant defenses. Fan et al. [14] reported that inoculation with endophytic bacterial strains L, K, and Y increased proline content and antioxidant enzyme activity in *Arabidopsis*, thereby promoting seedling growth under salt stress. Saline–alkali soils, such as those found in northeast China, present combined stress of high salinity and high pH, which differs fundamentally from pure salt stress. High pH not only exacerbates ion toxicity but also reduces the solubility and availability of phosphorus, iron and other micro-nutrients [5]. Therefore, endophytes suitable for saline–alkali conditions must possess traits that address both salinity and alkalinity, such as phosphate solubilization and siderophore production.

Endophytic bacteria offer several advantages over rhizosphere microorganisms for enhancing plant stress tolerance. First, endophytes colonize internal plant tissue, providing a more stable, protected niche that shields them from competitive exclusion by soil microbiota and from changes in soil pH, moisture, and temperature [7,15]. Second, their intimate association with plant cells allows for more efficient transfer of beneficial compounds, such as IAA and ACC deaminase, and for direct modulation of plant physiological responses [8]. Third, endophytes can systematically colonized the entire plant, potentially providing benefits to both roots and shoots through systemic induction of stress tolerance mechanisms [9]. Furthermore, endophytes isolated from plants growing in extreme environments are often better adapted to those conditions and may possess specialized traits not found in generalist rhizosphere bacteria.

Alfalfa (*Medicago sativa* L.) is a perennial forage legume known for its high protein content (15–22%), strong adaptability, and ability to improve soil fertility through biological nitrogen fixation, earning it the title of “King of Forages” [16,17]. It is cultivated on approximately 30 million hectares worldwide and serves as a critical feed source for livestock, with an estimated annual value of over $10 billion globally. Alfalfa exhibits moderate salt tolerance [18], and serious salt stress can adversely affect its growth and productivity [19], resulting in shorter, thicker roots with fewer lateral roots and root hairs, as well as reductions in fresh and dry biomass of 30–50% under severe stress [20]. To date, few studies have focused on screening endophytic bacterial resources from alfalfa and evaluating the growth-promoting potential of saline–alkali-tolerant strains. In this study, four endophytic bacterial strains were isolated from the roots of alfalfa growing in saline–alkali soils. These strains were identified using morphological characterization, 16S rDNA sequencing, and physiological–biochemical assays. Their ability to produce ACC deaminase, siderophores, and IAA, as well as solubilizing phosphorus, was analyzed. Pot and field experiments were subsequently conducted to evaluate their plant growth-promoting effects under saline–alkali conditions, laying the groundwork for developing alfalfa inoculants suitable for saline–alkali environments.

## 2. Materials and Methods

### 2.1. Sample Collection

Alfalfa plants were collected from the experimental base of the Heilongjiang Grassland Research Institute in Lanxi County, Harbin City, Heilongjiang Province, China (coordinates: 46°32′55.55″ N 126°01′41.62″ E). The plants were immediately placed in pre-sterilized bags to preserve freshness, stored in an icebox at 4 °C, and transported to the laboratory for the isolation and identification of saline–alkali-tolerant endophytic bacteria.

### 2.2. Isolation and Identification of Saline–Alkali Tolerant Endophytes

Root tissue samples (1 g) were surface-sterilized by soaking them in 75% ethanol for 3 min, followed by three rinses with sterile distilled water, treatment with 2% sodium hypochlorite for 30 s, and another three rinses with sterile water. Subsequently, the roots were placed on LB medium containing (per liter): 10 g of peptone, 5 g of yeast extract, 10 g of NaCl, and 15 g of agar. After surface drying, 2 mL of sterile water was added to the tissues in a sterile mortar and ground into a homogenate. Serial dilutions (10^−1^, 10^−2^, and 10^−3^) of the homogenate were prepared. Aliquots of each dilution were spread onto LB agar supplemented with 7% NaCl and adjusted to pH 9. Plates were incubated at 28 °C for 24–72 h. Well-isolated colonies with distinct morphologies were selected and repeatedly streaked onto fresh plates for purification over the 6th generation. Bacterial cultures were preserved in 50% sterile glycerol (*v*/*v*) with bacterial solution in a volume ratio of 1:1 at −80 °C for long-term storage. Bacterial genomic DNA was extracted using the CTAB/NaCl method [21]. The 16S rDNA gene was amplified by PCR using the universal bacterial primers F8 (5′-AGAGTTTGATCCTGGCTCAG-3′) and R1541 (5′-AAGGAGGTGATCCAGCCGCA-3′). The PCR products were purified and sent to Sangon Biotech (Shanghai, China) Co., Ltd., for DNA sequencing. The resulting sequences were compared with reference sequences in the GenBank database using BLAST (2.17.0). Multiple-sequence alignment and phylogenetic analysis were conducted in MEGA-X (10.2.1). A neighbor-joining phylogenetic tree was constructed with bootstrap. The 16S rDNA sequences obtained in this study have been deposited in the NCBI GenBank database under accession numbers SYM-2 (SUB16040103), SYM-4 (SUB16040197), SYM-15(SUB16040126), and SYM-9 (SUB16040051). Physiological and biochemical traits of the isolates were determined following the protocols outlined in Bergey’s Manual of Systematic Bacteriology [22].

### 2.3. Characterization of Plant Growth-Promoting Traits of Endophytes

#### 2.3.1. Determination of ACC Deaminase Activity

ACC deaminase activity was determined according to the method by Honma and Shimomura [23]. Strains were grown in ADF medium containing, per liter, 4 g of KH_2_PO_4_, 6 g of Na_2_HPO_4_, 0.2 g of MgSO_4_·7H_2_O, 2 g of glucose, 2 g of gluconic acid, 2 g of citric acid, 5.0 mmol of ACC, 0.01 mg of H_3_BO_3_, 0.0112 mg of MnSO_4_·H_2_O, 0.0778 mg of ZnSO_4_·7H_2_O, 0.05 mg of CuSO_4_·5H_2_O, 0.01 mg of MoO_3_, and 1.0 mg of FeSO_4_·7H_2_O. Cultures were incubated at 28 °C under shaking at 180 rpm for 48 h. The OD_540_ of the sample was measured and converted into α-ketobutyrate acid concentration by using a standard curve. One unit of ACC deaminase activity was defined as the amount of enzyme that produced 1 μmoL of α-ketobutyrate acid per minute under the assay conditions. Total protein content in the extracts was determined by the Bradford method [24]. ACC deaminase activity is expressed as μmol α ketobutyric acid mg^−1^ protein-h^−1^.

#### 2.3.2. Determination of Siderophore Production

Siderophore production was determined using the method by Schwyn and Neilands [25]. The strains were inoculated in MKB medium containing (per liter): 2.5 g of KH_2_PO_4_, 15 mL of glycerol, 5 g of acidified casein, and 2.5 g of MgSO_4_·7H_2_O. The samples were incubated at 28 °C under shaking at 180 rpm for 48 h. Cells were removed by centrifugation at 10,000× *g* for 5 min at 28 °C, and the supernatant was collected. The CAS assay solution was prepared as follow: solution A, 0.182 g of cetyltrimethylammonium bromide (CTAB) dissolved in 50 mL of deionized water; solution B, 0.0054 g of FeCl_3_·6H_2_O dissolved in 20 mL of 10 mM HCl; solution C, 0.0605 g of CAS dissolved in 50 mL of deionized water; solution D, 1.5 mL of solution B mixed with 7.5 mL of solution C, followed by addition of 6 mL of solution A; solution E, 4.307 g of piperazine-N,N′-bis (2-ethanesulfonic acid) (PIPES) completely dissolved in 20–30 mL of deionized water, with the addition of 6.25 mL of concentrated HCl and pH adjustment to 5.6. Solutions D and E were combined and diluted with deionized water to a final volume of 100 mL. For the assay, 0.5 mL of culture supernatant was mixed with 0.5 mL of CAS assay solution. After 1 h of incubation at room temperature in the dark, the absorbance at 630 nm (A) was measured. A control (Ar) consisted of 0.5 mL of deionized water mixed with 0.5 CAS assay solution. Siderophore production was expressed as the ratio A/Ar, where a lower ratio indicates higher siderophore activity.

#### 2.3.3. Determination of Phosphate Solubilization Ability

Phosphate solubilization was assessed following the method in [26] with modification. Strains were inoculated overnight in LB medium at 28 °C, and 1% (*v*/*v*) of each culture was them transferred to Pikovskaya’s (PVK) liquid medium containing (per liter): 10.0 g of glucose, 5.0 g of Ca_3_(PO_4_)_2_, 0.5 g of (NH_4_)_2_SO_4_, 0.3 g of MgSO_4_·7H_2_O, 0.3 g of KCl, 0.3 g of yeast powder, 0.3 g of NaCl, and 15.0 g of agar. Cultures were incubated at 28 °C under shaking at 180 rpm for 72 h. After incubation, cells were removed by centrifugation at 10,000× *g* for 10 min. The soluble phosphorus content in the supernatant was quantified using the molybdenum–antimony colorimetric method [27]. The amount of phosphate solubilized by each strain was calculated by subtracting the phosphorus content in an uninoculated control from the that in the sample.

#### 2.3.4. Determination of IAA Production

Indole-3-acetic acid (IAA) production was determined according to [28]. Isolates were initially grown in DF medium containing (per liter): 4 g of KH_2_PO_4_ 4, 6 g of Na_2_HPO_4_ 6, 0.2 g of MgSO_4_·7H_2_O, 2 g of glucose, 2 g of gluconic acid, 2 g of citric acid, 0.01 g of H_3_BO_3_, 0.0112 g of MnSO_4_·H_2_O, 0.0778 mg of ZnSO_4_·7H_2_O, 0.05 g of CuSO_4_·5H_2_O 0.05, 0.01 g of MoO_3_ 0.01, and 1.0 g of FeSO_4_·7H_2_O. After 48 h of incubation at 28 °C under shaking at 180 rpm, 1 mL of each culture was transferred to fresh DF medium supplemented with 0, 100, 200, and 500 µg·mL^−1^ L-tryptophan (L-Trp). These cultures were incubated for an additional 48 h under the same conditions. Bacterial growth was determined by measuring the OD at 600 nm (OD_600_). Cells were then pelleted by centrifugation at 10,000× *g* for 5 min at room temperature, and 500 µL of the supernatant was mixed with 2 mL of Salkowski’s reagent, which enables colorimetric detection of indolic compounds. After incubation in the dark at 28 °C for 30 min, the OD_535_ was measured. IAA content was determined using a standard curve prepared with pure IAA. All treatments were performed in triplicate.

### 2.4. Phosphorus and Fiber Determination

#### 2.4.1. Phosphorus Content Analysis

Phosphorus content in alfalfa was determined spectrophotometrically using the vanadomolybdate (yellow) method after wet acid digestion. Dried tissue samples were initially heated to 80–90 °C for 15–30 min to deactivate enzymes, followed by further drying at 60–70 °C until constant weight was achieved. A precisely weighted 0.10 g sample aliquot was digested with 5 mL of concentrated sulfuric acid in a 100 mL digestion tube. Following the initial reaction, sequential addition of 2 mL aliquots of 30% hydrogen peroxide (H_2_O_2_) was performed, with through shaking after each addition. The mixture was heated on a digestion block until complete dissolution of solids was observed, white fumes evolved, and the solution turned brown. Heating was temporarily halted; after brief cooling, 10 drops of H_2_O_2_ were added, and digestion continued for approximately 5 min. This cycle of cooling, H_2_O_2_ addition, and heating was repeated until the digest became clear. A final 5 min heating step ensured complete decomposition of residual H_2_O_2_. The cooled digest was quantitatively transferred into a 100 mL volumetric flask and diluted to volume. A reagent blank was processed concurrently. The standard curve for phosphorus showed excellent linearity, with R^2^ = 0.9919. For colorimetric analysis, 10 mL of the digest was transferred to a 50 mL flask and tested identically to the standards, beginning from the diluting to about 30 mL with a water step. Absorbance was measured at 450 nm using the reagent blank as the reference. Finally, phosphorus content (g/kg) was calculated.

#### 2.4.2. Neutral Detergent Fiber (NDF) Analysis

Neutral detergent fiber content was determined following Chinese National Standard GB/T 20806-2006 [29]. Approximately 0.50 g of sample was weighted in a 600 mL tall-form beaker. Subsequently, 100 mL of neutral detergent solution and 2–3 drops of 1-octanol (as an anti-foaming agent) were added. The natural detergent solution contained (per liter): 18.61 g of disodium EDTA dihydrate, 6.81 g of sodium borate decahydrate, 30 g of sodium lauryl sulfate, 10.0 mL of triethylene glycol, 4.56 g of disodium hydrogen phosphate anhydrous, and 10.0 mL of ethanol (95%). The solution pH was adjusted to 6.9–7.1. The mixture was heated under reflux, rapidly brought to a boil, and maintained at gentle boiling for exactly 60 min from the onset of boiling. Prior to filtration, a sintered glass funnel with pore size 40–60 μm was dried at 105 °C to constant weight and its mass recorded. The hot digest was immediately filtered through the pre-weighted funnel under vacuum. The residue was thoroughly washed with hot water (90–100 °C) until the filtrate was clear and free of foam, followed by three successive rinses with acetone to ensure complete removal of residual water and solubles. The funnel containing the residue was then dried at 105 °C for 3–4 h until constant weight was achieved, cooled in desiccator, and weighted. The drying (30 min), cooling, and weighing cycle was repeated until mass variation was negligible. Finally, NDF content (%) was calculated.

#### 2.4.3. Acid Detergent Fiber (ADF) Analysis

Acid detergent fiber content was analyzed in accordance with Chinese Agricultural Industry Standard NY/T 1459-2007 [30]. The acid detergent solution contained cetyltrimethylammonium bromide (CTAB) dissolved in 1 N sulfuric acid (prepared by adding 27.8 mL of concentrated H_2_SO_4_ to distilled water and bringing the volume to 1 L). Clean sintered glass filtering crucibles were conditioned by drying at 105 °C for 4 h, cooled in a desiccator for 30 min, and weighted; this process was repeated until constant weight was attained. Exactly 1.00 g of sample was digested in 100 mL of preheated acid detergent solution under reflux for 60 min, with the heat being adjusted to maintain gentle boiling throughout. The digest was filtered under vacuum through a pre-weighted crucible. The collected residue was broken up with a glass rod and washed 3–5 times with hot water (90–100 °C) to ensure complete acid removal, followed by two washes with approximately 40 mL of acetone (3–5 min soaking per wash). After allowing residual acetone to evaporate under a fume hood, the crucible with residual was dried at 105 °C for 4 h, cooled in a desiccator for 30 min, and weighted. The drying–cooling–weighing cycle was repeated until constant weight was achieved. Finally, ADF content (%) was calculated.

### 2.5. Planting of Alfalfa

#### 2.5.1. Pot Experiment

The seeds of alfalfa were provided by the Animal Husbandry Branch of the Heilongjiang Provincial Academy of Agricultural Sciences. The test saline–alkali soil (pH 8.40; EC, 288 μs·cm^−1^; organic matter, 28.19 g·kg^−1^; alkali-hydrolyzable nitrogen, 121.57 mg·kg^−1^; available phosphate, 25.7 mg·kg^−1^; available potassium, 121.73 mg·kg^−1^) was collected from Shengli Village, Yuanda Town, Lanxi County, Suihua City, Heilongjiang Province. The soil was passed through the 1 mm mesh sieve to remove weeds and gravel. Each pot was filled with 0.75 kg of soil. The isolated SYM-2, SYM-4, SYM-9, and SYM-15 strains were inoculated into LB liquid medium and cultured under the 28 °C, 180 r·min^−1^ condition. Next, centrifugation was carried out to collect the bacterial precipitate. The bacteria were re-suspended in sterile water to achieve the concentration of 1 × 10^8^ cfu·mL^−1^, thereby preparing the bacterial solution. The surface of alfalfa seeds was disinfected (75% alcohol disinfection for 1 min, rinsing with sterile water 2 times, disinfection with 1% hypochlorous acid solution for 10 min, and rinsing with sterile water 3 times). The control and bacterial inoculation treatments were set up. The seeds were soaked in SYM-2, SYM-4, SYM-9, and SYM-15 bacterial suspensions (1 × 10^8^ cfu·mL^−1^) for 2 h, and seeds that soaked in sterile water served as the control treatment. The seeds of the inoculation and control treatments were sown in the pots, with 5 seeds in each pot, which was repeated 3 times. Then, they were placed in the greenhouse (24 °C, 16 h/8 h light/dark) for cultivation. During the growth process of alfalfa, 50 mL of bacterial suspension was evenly applied around the roots of the seedlings every 7 d, and the control used the same amount of sterile water instead.

#### 2.5.2. Field Experiment

The field trail was conducted at the Lanxi Base of Grassland Branch of Heilongjiang Academy of Agricultural Sciences (N 46°12′, E 126°08′, mean altitude of 160 m). Soil characteristics were pH 8.40 and EC of 288 μs·cm^−1^. Strain SYM-15 was prepared in LB medium, incubated overnight, and diluted to 1 × 10^8^ cfu·mL^−1^ with sterile water. Seeds were soaked in the bacterial suspension for 2 h (control seeds were placed in an equal volume of sterile water) and then shade-dried. For each treatment, 3 g of seed was sown evenly in 1 m × 1 m plots, with four replicate plots per treatment. At 30-day intervals, each plot received 1 L of the bacterial suspension (controls group received sterile water) through gentle soil drenching around the plants.

### 2.6. Determination of Plant Growth

After 4 weeks of incubation, the leaves of potted alfalfa were collected, separated, and stored at −80 °C. Biomass of potted alfalfa was measured after 8 weeks of incubation. Root vigor was determined using the 2,3,5-triphenyltetrazolium chloride (TTC) method [31]; soluble protein and free proline contents were determined using established methods [32,33]; peroxidase and superoxide dismutase activities were determined by the guaiacol oxidation method and the nitrogen blue tetrazolium (NBT) photo-reduction method, respectively [34]. Hydrogen peroxide content, catalase activity, thiobarbituric acid (TBA), and hydroxylamine oxidation methods were used to determine plant malondialdehyde and superoxide anion contents [35,36,37]. After harvest, alfalfa yield was recorded, and protein content was determined using the Kjeldahl method [38].

### 2.7. Statistical Analysis

Experimental data were tabulated using Microsoft Excel 2022 (Microsoft Corp., Redmond, WA, USA) and Origin 2022 and statistically analyzed using SPSS 26.0 (IBM, Chicago, IL, USA). One-way analysis of variance (ANOVA) and *t*-tests were performed to compare data between treatment groups.

## 3. Results

### 3.1. Isolation and Identification of Saline–Alkali-Tolerant Endophytes

Four endophytic bacterial strains capable of growing under saline–alkali conditions were isolated from alfalfa root tissues collected from saline–alkali soil. After purification, the isolates were designated SYM-2, SYM-4, SYM-9, and SYM-15. Strain SYM-2 formed round, opaque-white colonies with dry surface, flat center, and uneven edges. Strains SYM-4, SYM-9, and SYM-15 produced circular, moist colonies with raised center; SYM-9 and SYM-15 colonies were semi-transparent and exhibited smooth edges (Table S1).

**Figure 1:**
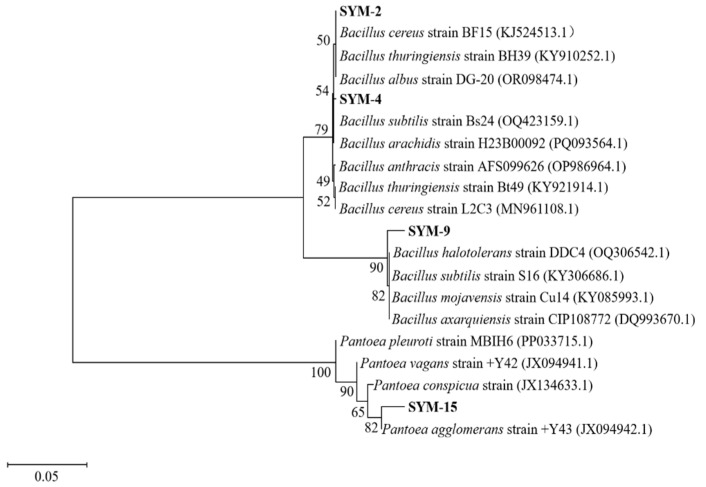
Phylogenetic tree of bacterial strains based on 16S rDNA sequencing.

The resulting sequences were compared with reference sequences in the NCBI “GenBank” database using BLASTn (2.17.0). Strain SYM-2 showed high homology (99.86%) with *Bacillus cereus* and 99.73% with *B. thuringiensis.* Strain SYM-4 exhibited 99.66% similarity to *B. thuringiensis*, 99.60% to *B. cereus*, and 99.80% to *B. arachidis*. Strain SYM-9 displayed 99.65% homology to *B. halotolerans*, 99.59% to *B. subtilis*, 99.65% to *B. mojavensis*, and 99.59% to *B. axarquiensis*. Strain SYM-15 was closely related to *Pantoea agglomerans* (99.01%)*, P. vagans* (98.40%), *P. conspicua* (97.90%), and *P. pleuroti* (97.66%). Phylogenetic analysis based on the 16S rDNA sequences confirmed that strains SYM-2, SYM-4, and SYM-9 belonged to the genus *Bacillus*, while SYM-15 belonged to the genus *Pantoea* (Figure 1).

Physiological and biochemical tests further distinguished the four isolates (Table 1). Strains SYM-2, SYM-4, and SYM-9 were oxidative-negative, methyl red-negative, indole-negative, gelatin liquefaction-negative, and hydrogen sulfide-negative. All three were positive for Voges–Proskauer (VP) reaction, starch hydrolysis, and Gram staining. Strains SYM-4 and SYM-9 utilized sucrose, whereas SYM-2 did not. Strains SYM-2 and SYM-4 were citrate-negative, while SYM-9 was citrate-positive. In contrast, SYM-15 was positive for sucrose, methyl red, Voges–Proskauer, citrate, indole, and gelatin liquefaction but negative for oxidase, starch hydrolysis, hydrogen sulfide, and Gram staining. Combined with colony morphology and 16S rDNA-based phylogenetic analysis (Figure 1, Table S1), SYM-2, SYM-4, SYM-9, and SYM-15 were identified as closely related to *B. cereus*, *B. thuringiensis*, *B. halotolerans*, and *P. agglomerans*, respectively.

### 3.2. Plant Growth-Promoting Traits of Endophytes

**Figure 2:**
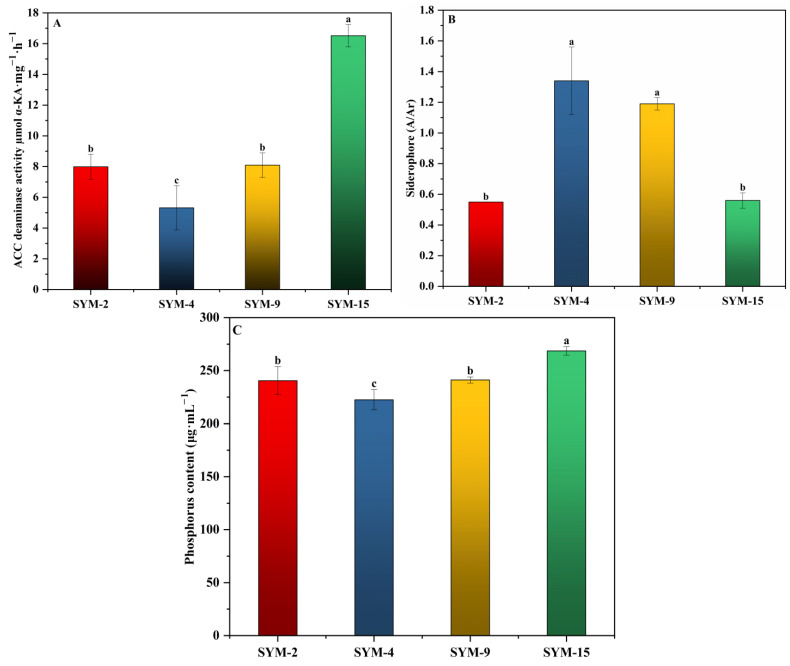
Plant growth-promoting traits of isolated bacterial strains. ACC deaminase activity (**A**), siderophore production (**B**), and phosphorus solubilization (**C**). Different letters above bars represent significant differences among strains at *p* < 0.05, and the same letter represents non-significant differences according to one-way ANOVA followed by Duncan’s multiple range test (*p* < 0.05).

The four endophytic strains were evaluated for key plant growth-promoting traits, including ACC deaminase activity, siderophore production, phosphorus solubilization, and IAA production. ACC deaminase activity ranged from 5.32 to 16.52 µmol α-ketobutyric acid mg^−1^ protein-h^−1^. SYM-15 showed the highest activity (16.52), followed by SYM-9 (8.10), SYM-2 (7.99), and SYM-4 (5.32) (Figure 2A). In the CAS assay, siderophore production is indicated by a decrease in A/Ar ratio, with lower values indicating higher siderophore activity. SYM-2 (A/Ar = 0.55) and SYM-15 (A/Ar = 0.56) showed strong siderophore production, while SYM-4 (A/Ar = 1.34) and SYM-9 (A/Ar = 1.19) showed minimal or no siderophore activity, as values > 1 indicate no detectable siderophore production (Figure 2B). Phosphorus solubilization was quantified as soluble phosphorus released into the medium. The effective phosphorus content ranged from 222.49 to 268.62 μg·mL^−1^, with SYM-15 solubilizing the most phosphorus (268.62 μg·mL^−1^) (Figure 2C). IAA production was assessed at different L-tryptophan concentrations (0–500 μg·mL^−1^). All strains synthesized IAA, and production increased with higher tryptophan supply. At 500 μg·mL^−1^ tryptophan, IAA yields were 5.65 (SYM-2), 10.37 (SYM-4), 7.06 (SYM-9), and 11.10 μg·mL^−1^ (SYM-15) (Table 2). No toxicity was observed at the highest tryptophan concentration, as bacterial growth was not inhibited.

### 3.3. Growth-Promoting Effect of Endophytes in Alfalfa

In the pot experiment, compared with the control treatment, the plant height of alfalfa after inoculation with SYM-2, SYM-4, SYM-9, and SYM-15 increased by 4.07%, 17.38%, 27.34%, and 33.90%, respectively; and the root length increased by 7.49%, 10.05%, 22.20%, and 27.94%, respectively (Table 3). In terms of the fresh weight of the shoots, compared with the control treatment, inoculation with SYM-2, SYM-4, SYM-9, and SYM-15 increased it by 3.70%, 34.26%, 45.37%, and 49.07%, respectively. In terms of the dry weight of the shoots, inoculation with SYM-2, SYM-4, SYM-9, and SYM-15 resulted in increases of 7.41%, 29.63%, 51.85%, and 59.26%, respectively, compared with the control treatment. Therefore, this study also demonstrated similar increasing trends in the fresh/dry weight of alfalfa roots (Table 3).

**Figure 3:**
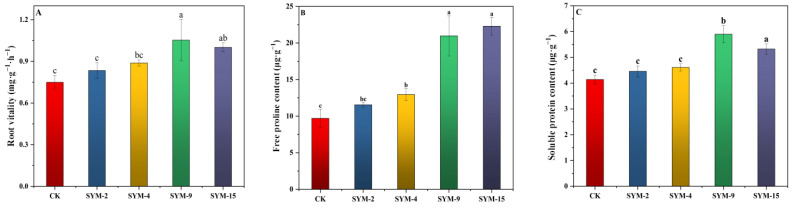

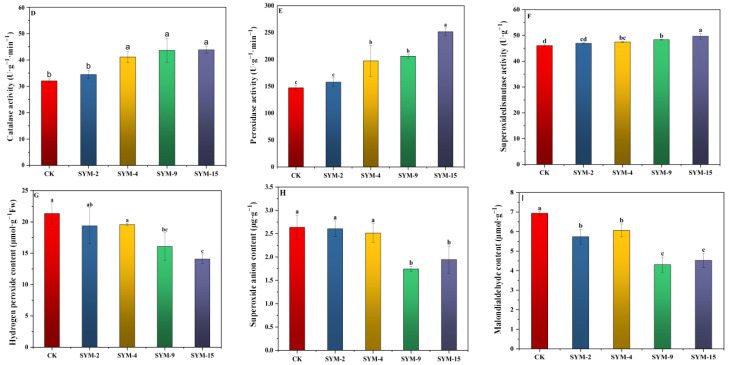
Effects of bacterial strain inoculation on the physiological characteristics of alfalfa under saline–alkali conditions. (**A**) Root activity, (**B**) free proline content, (**C**) soluble protein content, (**D**) catalase activity, (**E**) peroxidase activity, (**F**) superoxide dismutase activity, (**G**) hydrogen peroxide content, (**H**) superoxide anion content, and (**I**) malondialdehyde content. Different letters above bars represent significant differences among strains at *p* < 0.05, and the same letter represents non-significant differences according to one-way ANOVA followed by Duncan’s multiple range test (*p* < 0.05).

In the pot experiment, inoculation with the endophytic strains significantly improved root activity and osmotic adjustment, increased antioxidant enzyme activity, and reduced oxidative damage in alfalfa grown under saline–alkali conditions compared with the control (*p* < 0.05). Root activity increased by 11.23% (SYM-2), 18.44% (SYM-4), 40.38% (SYM-9), and 33.48% (SYM-15) (Figure 3A). Proline content increased by 19.09–129.87% across the inoculated treatments (Figure 3B). Soluble protein content was increased by 7.71% (SYM-2), 11.39% (SYM-4), 42.49% (SYM-9), and 28.61% (SYM-15) (Figure 3C). Catalase (CAT) activity increased by 7.48–36.70%, peroxidase (POD) by 7.15–70.77%, and superoxide dismutase (SOD) by 1.91–7.86% relative to the control (Figure 3D–F). Hydrogen peroxide (H_2_O_2_) content increased by 8.27–34.00% (Figure 3G) and superoxide anion (O_2_^−^) content by 1.29–33.89% (Figure 3H). Malondialdehyde (MDA) content, an indicator of lipid peroxidation, was reduced by 17.31% (SYM-2), 12.62% (SYM-4), 37.94% (SYM-9), and 34.77% (SYM-15) (Figure 3I). In the pot experiment, compared with the control and other treatments, including SYM-9, peroxidase activity and superoxide dismutase activity in alfalfa significantly increased after the SYM-15 treatment, while hydrogen peroxide content significantly decreased. This indicates that inoculation with SYM-15 can significantly improve the physiological characteristics of alfalfa, helping plants to resist salt and alkali stress.

**Figure 4:**
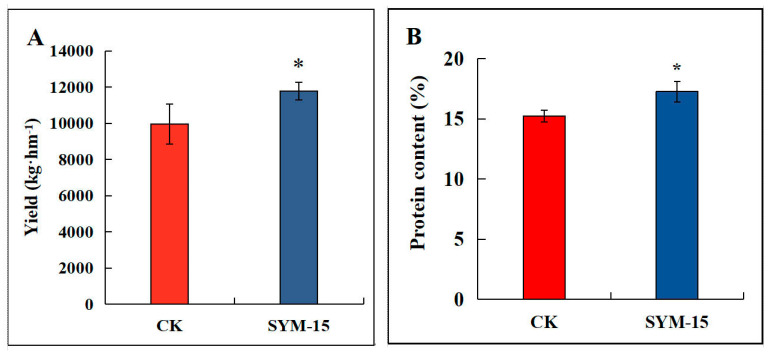

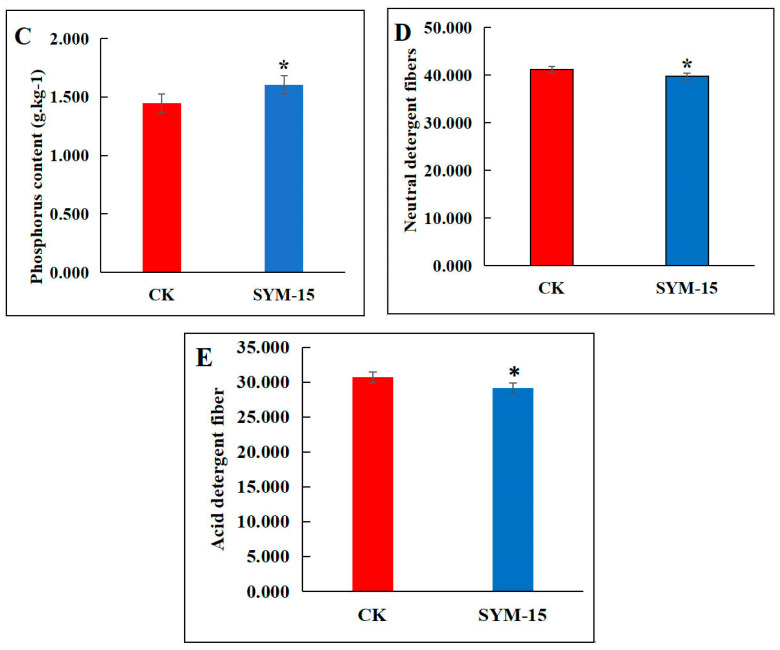
Effects of isolates on plant yield (**A**), protein content (**B**), phosphorus content (**C**), neutral detergent fiber (**D**), and acid detergent fiber (**E**) in alfalfa in the field experiment. Different asterisks (*) above bars represent significant differences between CK and SYM-15 treatments according to *t*-test (* *p* < 0.05).

Based on its superior growth-promoting performance in the pot experiment, strain SYM-15 was selected for the field evaluation. Inoculation with SYM-15 significantly increased the yield of and crude protein content in alfalfa compared with the uninoculated control (*p* < 0.05). Yield increased by 34.55% (Figure 4A), and crude protein content increased by 13.35% (Figure 4B). In the field experiments, after inoculation with SYM-15, phosphorus content in alfalfa significantly increased by 11.09% (Figure 4C), while neutral detergent fiber and acid detergent fiber significantly decreased by 3.43% and 5.06% respectively (Figure 4D,E). These results show that endophytic strain SYM-15 effectively alleviated the negative impact of saline–alkali stress on alfalfa growth and substantially improved both productivity and forage quality under field conditions.

## 4. Discussion

### Isolation and Identification of Saline–Alkali Tolerant Endophytes

Saline–alkali stress is a major abiotic constraint that severely limits plant survival and productivity [39]. In this context, microbial inoculation has emerged as a promising bioremediation approach to improve crop resilience and support sustainable agriculture in salt-affected soil. Previous studies have highlighted the potential of plant growth-promoting bacteria (PGPB) for mitigating salt stress. Ma et al. [40] isolated four PGPR strains (*Priestia*, *Bacillus*, and *Paenibacillus* spp.) capable of producing ACC deaminase and IAA; these strains enhanced alfalfa seedlings growth and flowering under salinity. Similarly, Khoso et al. [41] reported that rhizobacterium *B. subtilis* strain AD13-4, isolated from saline–alkali soil, significantly increased plant biomass under stress conditions. In line with these findings, the present study isolated four endophytic bacterial strains from alfalfa roots grown in saline–alkali soil. Through polyphasic identification, including colony morphology, 16S rDNA sequencing, and physiological–biochemical profiling, strains SYM-2, SYM-4, SYM-9, and SYM-15 were identified as closely related to *B. cereus*, *B. thuringiensis*, *B. halotolerans*, and *P. agglomerans*, respectively. These strains showed strong growth-promoting effects on alfalfa in saline–alkali environments and thus hold promise for developing microbial agents for saline–alkali-tolerant alfalfa.

Endophytic bacteria possess a range of beneficial biological traits, such as IAA production, siderophore production, phosphorus solubilization, and ACC deaminase activity, that promote plant development and increase resistance to abiotic stress [42]. The four strains isolated in this study exhibited these key PGP attributes, suggesting their potential support of alfalfa growth under saline–alkali conditions. Similar PGP mechanisms have been reported in other stress-tolerant endophytes. The ACC deaminase activity of SYM-15 (16 μg·mL^−1^·h^−1^) is notable compared with previously reported salt-tolerant endophytes. For example Lu et al. [12] isolated *P. ananatis* D1 from rice, which enhanced root and shoot growth under salinity through IAA synthesis, siderophore release, and phosphorus mobilization. Likewise, Bokhari et al. [43] demonstrated that endophytic *B subtilis* from desert plants improved salt tolerance in *Arabidopsis* via analogous traits. Our findings align with these studies, reinforcing the role of multiple PGP traits in host cross-stress mitigation. ACC deaminase activity is particularly important for reducing ethylene-mediated growth inhibition under stress [44]. All four strains in this study produced ACC deaminase, with SYM-15 showing notably high activity (16.52 µmol α-KA·mg^−1^·h^−1^). This is consistent with reports of ACC deaminase-positive endophytes from plants [45]. Lu et al. [12] reported 4.8–15.3 µmol·mg^1^·h^−1^ for rice endophytes, which supports the hypothesis that our strains alleviate saline–alkali stress partly by modulating ethylene levels in alfalfa.

Saline–alkali stress typically reduces plant growth rate and biomass accumulation; however, inoculation with stress-tolerant endophytes can counteract these effects. For instance, Siddiqui et al. [46] demonstrated that halophyte-derived endophytes significantly increased rice and maize biomass under 150 mM NaCl-induced stress. Similarly, Yang et al. [47] reported that *Burkholderia phytofirmans* improved quinoa growth under salinity, while Kumari et al. [48] observed enhanced root development and biomass in soybean inoculated with *Pseudomonas* sp. AK-1 and *Bacillus* sp. SJ-5 under salt stress. Our study showed that the four considered endophytic strains significantly increased alfalfa plant height, root length, and fresh and dry biomass under saline–alkali conditions (Table 3), confirming them as effective growth promoters under stress.

In addition to promoting growth, endophytes enhance plant stress tolerance by stimulating osmoregulatory responses. Proline and soluble protein are key osmoregulatory solutes that accumulate under abiotic stress to maintain cellular balance and mitigate damage [49,50,51]. PGPR can accelerate the synthesis of these compounds, thereby improving the host plant’s osmotic adjustment capacity [52]. In our study, inoculation with endophytic bacterial strains significantly increased both protein and soluble protein contents in alfalfa in saline–alkali environments (*p* < 0.05; Figure 3B,C), indicating active contribution to osmotic homeostasis and stress resistance. Saline–alkali stress also leads to overproduction of reactive oxygen species (ROS), such as superoxide anions and hydrogen peroxide, which can oxidize lipid proteins and nucleic acids, leading to membrane damage and cytotoxicity [53]. Plants counteract ROS through the enzymatic activity of antioxidants including SOD, POD, and CAT [54]. Endophytic bacteria are known to enhance plant antioxidants capacity and ROS-scavenging systems, thereby improving salt tolerance [55].

For example, Dif et al. [56] reported that endophytic inoculation upregulated antioxidation-related genes and reduced ROS levels in tomato under salt stress, while Abd Allah et al. [57] observed increased SOD, POD, and CAT activities in chickpea inoculated with *B. subtilis* BERA71 under salinity. Our results align with these reports: Inoculation with the four strains increased POD, CAT, and SOD activities in alfalfa (Figure 3D–F) while simultaneously reducing H_2_O_2,_ O_2_^−^, and MDA contents (Figure 3G–I). The similar biomass increases with SYM-9 and SYM-15 (Table 3), despite SYM-9 having lower ACC deaminase activity and no detectable siderophore production, suggests that different combinations of PGP traits can achieve comparable growth promotion. However, the superior physiological responses to SYM-15 (higher POD and SOD; lower H_2_O_2_) indicate that SYM-15 provides additional benefits for stress alleviation that may become more important under severe stress conditions. In addition, strain SYM-15 demonstrated outstanding performance under field conditions, as it increased alfalfa yield, and protein and phosphorus contents in saline–alkali field soil (Figure 4). Inoculation with endophytes SYM-15 enhanced alfalfa quality by improving both biomass and nutritional content, increasing both yield and protein by 34.55% and 13.35%, respectively (Figure 4A,B). The 13.35% increase in crude protein content with SYM-15 compares favorably with previous reports indicating 5–15% protein increases with microbial inoculation in forage crops [56]. The reduction in fiber contents (NDF and ADF) with bacterial inoculation (Figure 4D,E) has important implications for forage quality. Under stress conditions, plants often increase lignin deposition as a defense mechanism. The stress alleviation provided by SYM-15 likely reduced the need for such stress-induced lignification, allowing for more resources and increasing protein content (Figure 4B) and for the strong negative correlation between protein and fiber contents across treatments (r = 0.82, *p* < 0.01). Furthermore, phosphorus, essential to photosynthesis and forage quality, can be increased by phosphate-solubilizing bacteria such as Erwinia, Rhizobium, Bacillus, and Pseudomonas [57]. Similarly, in our study, phosphorus content in inoculated alfalfa reached 1.61 g/kg, significantly exceeding the control. Fiber content critically affects protein digestibility in herbivores. PGPR inoculation was shown to reduce neutral and acid detergent fiber in alfalfa (104). Similarly, in our endophyte inoculation, particularly SYM-15 significantly decreased these fiber components, enhancing overall forage quality (Figure 4). This indicates that the endophytes effectively bolstered the antioxidant defense system and attenuated damage in alfalfa under saline–alkali conditions.

## 5. Conclusions

Four saline–alkali-tolerant endophytic bacterial strains (SYM-2, SYM-4, SYM-9, and SYM-15) were isolated from alfalfa roots growing in saline–alkali soil in northeast China. Based on 16S rDNA sequencing and biochemical characterization, these strains were identified as closely related to *B. cereus*, *B. thuringiensis*, *B. halotorans*, and *Pantoea agglomerans*, respectively. All four exhibited key plant PGP traits, including ACC deaminase activity, IAA production, phosphorus solubilization and (for SYM-2 and SYM-15) siderophore production. Under saline–alkali conditions, inoculation with these strains significantly improved alfalfa growth, enhancing plant height, root length, and both above-/below-ground biomass. Inoculation also increased root activity, the accumulation of osmoregulatory substances (proline and soluble protein) and antioxidant enzymatic activity (CAT, POD, and SOD), while reducing oxidative damage markers (H_2_O_2_, O_2_^−^, and MDA). Compared with the control and other treatments, including SYM-9, the peroxidase activity and superoxide dismutase activity of alfalfa significantly increased after the SYM-15 treatment, while hydrogen peroxide content significantly decreased. Notably, strain SYM-15 demonstrated outstanding performance under field conditions, as it significantly increased alfalfa yield, and protein and phosphorus contents in saline–alkali field soil. On the other hand, after inoculation, neutral detergent fiber and acid detergent fiber contents decreased. The isolated endophytes, especially SYM-15, effectively enhance alfalfa growth and stress tolerance through multiple PGP mechanisms, positioning them as valuable strains for developing microbial inoculants to improve alfalfa productivity in saline–alkali-affected agriculture.

This study makes several contributions: (1) it identifies endophytic bacteria from alfalfa adapted to extreme saline–alkali conditions (pH 9, 7% NaCl); (2) it provides a comparative analysis of strains with different PGP trait combinations; (3) it validates pot experiment findings with field trials; and (4) it suggests that growth promotion may be achieved through different mechanistic pathways, with SYM-15 combining multiple alkaline-specific traits.

## Figures and Tables

**Table 1:** Physiological and biochemical characteristics of the studied strains.

Index	SYM-2	SYM-4	SYM-9	SYM-15
Oxidase	−	−	−	−
Sucrose	−	+	+	+
Methyl red	−	−	−	+
Voges–Proskauer	+	+	+	+
Citrate	−	−	+	+
Indole	−	−	−	+
Starch	+	+	+	−
Gelatin liquefaction	−	−	−	+
H_2_S	−	−	−	−
Gram staining	+	+	+	−

Note: “+” represents the existence of the corresponding ability; “−” indicates the absence of the corresponding ability.

**Table 2:** IAA production ability of isolated bacterial strains.

Strain	IAA IAA Content (μg·mL^−1^)			
	Without L-Tryptophan	With 100 μg·mL^−1^ L-Tryptophan	With 200 μg·mL^−1^ L-Tryptophan	With 500 μg·mL^−1^ L-Tryptophan
SYM-2	4.01 ± 0.38 d	4.24 ± 0.44 d	4.88 ± 0.29 c	5.65 ± 0.09 d
SYM-4	5.86 ± 0.16 b	6.15 ± 0.47 b	9.10 ± 0.32 a	10.37 ± 0.12 b
SYM-9	4.66 ± 0.36 c	5.10 ± 0.44 c	6.26 ± 0.31 b	7.06 ± 0.29 c
SYM-15	7.18 ± 0.25 a	8.34 ± 0.09 a	8.88 ± 0.20 a	11.10 ± 0.13 a

Different lowercase letters within the same column indicate significant differences among treatments at *p* < 0.05, while the same letter indicates no significant differences according to one-way ANOVA followed by Duncan’s multiple range test (*p* < 0.05).

**Table 3:** Effects of bacterial strain inoculation on alfalfa biomass under saline–alkali conditions.

Treatment	Plant Height (cm)	Root Length (cm)	Fresh Weight of Shoots (g/5 Plants)	Dry Weight of Shoots (g/5 Plants)	Fresh Weight of Roots (g/5 Plants)	Dry Weight of Roots (g/5 Plants)
CK	15.25 ± 0.68 c	14.82 ± 0.69 b	1.08 ± 0.11 b	0.27 ± 0.03 c	0.88 ± 0.12 c	0.18 ± 0.03 b
SYM-2	15.87 ± 0.65 c	15.93 ± 0.67 b	1.12 ± 0.07 b	0.29 ± 0.09 c	0.92 ± 0.04 c	0.19 ± 0.03 b
SYM-4	17.90 ± 0.66 b	16.31 ± 1.13 b	1.45 ± 0.13 a	0.35 ± 0.05 b	1.11 ± 0.03 bc	0.25 ± 0.02 a
SYM-9	19.42 ± 1.05 ab	18.11 ± 0.82 a	1.57 ± 0.14 a	0.41 ± 0.03 a	1.29 ± 0.02 ab	0.29 ± 0.04 a
SYM-15	20.42 ± 1.14 a	18.96 ± 0.86 a	1.61 ± 0.06 a	0.43 ± 0.07 a	1.28 ± 0.09 a	0.30 ± 0.04 a

Different lowercase letters within the same column indicate significant differences among treatments at *p* < 0.05, while the same letter indicates no significant differences according to one-way ANOVA followed by Duncan’s multiple range test (*p* < 0.05).

## Data Availability

The entire data presented in this study are available upon request from the corresponding author.

## Supplementary Materials

The following supporting information can be downloaded at: https://www.mdpi.com/article/10.3390/biology15060474/s1, Table S1: Colony morphology of the isolated strains.
