# Supplementary material for: The Application of Saline–Alkali-Tolerant Growth-Promoting Endophytic Bacteria for Enhancing the Saline–Alkali Tolerance of Alfalfa
Source: Biology (Basel). 2026 Mar 15;15(6):474. doi: 10.3390/biology15060474 (PMC13023798; doi:10.3390/biology15060474)
Supplement: Supplementary file 1 [file biology-15-00474-s001.zip › biology-4197733-supplementary.pdf]

Table S1. Colony morphology of the isolated strains.

Strains	Colony Morphology					
	**Shape**	**Drying/Wetting**	**Flattening/Bulging**	**Transparency**	**Color**	**Neat/Uneven Edges**
SYM-2	Round	Dry	Flatten	Opaque	White	Uneven
SYM-4	Round	Moist	Bulge	Opaque	Off-white	Uneven
SYM-9	Round	Moist	Bulge	Semi transparent	White	Neat
SYM-15	Round	Moist	Bulge	Semi transparent	Yellow	Neat
